# Safety of the primary percutaneous coronary intervention strategy combining pre-hospital prasugrel, enoxaparin and in-hospital bivalirudin in acute ST-segment elevation myocardial infarction

**DOI:** 10.1186/s12872-016-0333-0

**Published:** 2016-07-30

**Authors:** Juho Viikilä, Tuomo Nieminen, Ilkka Tierala, Mika Laine

**Affiliations:** 1 Department of Cardiology, Päijät-Häme Central Hospital, Keskussairaalankatu 7, 15850 Lahti, Finland; 2 Department of Cardiology, Helsinki University Central Hospital, Helsinki, Finland

## Abstract

**Backround:**

The optimal antithrombotic treatment during a primary percutaneous coronary intervention (pPCI) is not known. This single center registry study aims to assess the safety of a novel antithrombotic regimen combining enoxaparine and prasugrel at presentation, followed by bivalirudin at the catheterisation laboratory.

**Methods:**

All consecutive patients who underwent a pPCI were collected prospectively. The primary endpoint was major bleeding within 30 days. The secondary endpoints were a composite of major adverse cardiovascular events (MACE) consisting of cardiovascular death, non-fatal myocardial infarction, non-fatal stroke, a new target vessel revascularisation and all-cause mortality at 30 days.

**Results:**

Ninety-nine out of the total of 390 patients were treated according to the new regimen (protocol-treated group). The rest received other antithrombotic treatment (non-protocol-treated group). The protocol-treated group had a lower risk than the non-protocol-treated group according to the GRACE ischaemic (112 vs. 124, *p* = 0.002) and CRUSADE bleeding scores (21 vs. 28, *p* < 0.0001). The incidences of bleeding were similar: severe GUSTO or TIMI bleeding occurred in 0 % of the protocol-treated group and in 1.0 and 0.3 %, respectively, of the other group (*p* = 0.311 for GUSTO and *p* = 0.559 for TIMI). The incidence of MACE in the groups was 6.1 and 10.7 %, respectively (*p* = 0.178). The respective incidences of all-cause mortality were 5.1 and 9.6 % (*p* = 0.158).

**Conclusions:**

Administration of the novel antithrombotic regimen seems to be safe.

## Background

Primary percutaneous coronary intervention (pPCI) is the preferred first-line treatment for acute ST- segment elevation myocardial infarction (STEMI) [1]. Antithrombotic treatment is an essential part of the pPCI procedure in enhancing the opening of the occluded coronary artery as well as preventing peri- and post-procedural thrombotic complications and late recurrent ischaemic events. Peri-procedural stroke, for example, has been recognised as an important life-limiting complication [2]. The development of new antithrombotic agents has been rapid over the last decade. The application of clopidogrel and glycoprotein (GP) IIb/IIIa inhibitors in addition to heparin and aspirin has been shown to decrease early and late adverse cardiac events with a concomitant and undesired increase in bleeding events [3–6]. Bleeding has been recognised as a major determinant of cardiovascular death and adverse events in acute coronary syndrome (ACS) patients [7].

Prasugrel is a novel thrombocyte receptor P2Y12 inhibitor, which has a more rapid, efficacious and consistent antithombotic effect than clopidogrel [8, 9]. In the TRITON TIMI-38 trial, prasugrel was more effective than clopidogrel in reducing adverse cardiac events in both acute non-ST-elevation myocardial infarction and STEMI patients [10]. However, the incidence of severe bleeding events, especially in elderly and low-weight patients as well as those with prior ischaemic cerebrovascular events was raised with prasugrel when compared to clopidogrel.

Bivalirudin is an intravenously administered direct, short-acting thrombin inhibitor [11]. In the HORIZONS-AMI trial, it reduced severe bleeding events as well as early and late net adverse events in STEMI patients when compared to heparin plus GPIIb/IIIa-inhibitor, with an increase in the incidence of early stent thromboses [12]. In the EUROMAX study, bivalirudin started during transport for pPCI was similarily associated with a reduction in major bleeding events and an increase in early stent thrombosis compared to heparin or enoxaparin with optional GPIIb/IIIa inhibitor [13]. The recent HEAT-PPCI compared heparin and bivalirudin in a randomised settings; heparin reduced the incidence of major ischaemic events with equal safety profile [14]. Importantly, the other antithrombotic drugs used differed between the studies.

Enoxaparine is an alternative to unfractionated heparin. In the ATOLL trial, intravenous enoxaparine, in comparison to heparin, was associated with fewer ischaemic events with similar bleeding rates for acute STEMI patients undergoing pPCI [15].

No previous study has combined prehospital prasugrel and enoxaparine with bivalirudin. We hypothesized that using this combination of drugs might balance the risk of early stent thrombosis and bleeding events in pPCI patients. Thus, we report the results of our new antithrombotic pPCI regimen including aspirin, a low-dose enoxaparine i.v. bolus and prasugrel loading upon first medical contact (FMC) combined with a bivalirudin infusion initiated in the catheterisation laboratory.

## Methods

### Study patients and data collection

The present study took place in the Meilahti hospital, responsible of all PCIs for STEMI in the Helsinki-Uusimaa Hospital District of 1.5 million inhabitants. According to the new local STEMI guidelines launched on 1 November 2010, all acute STEMI patients referred to pPCI should receive aspirin 250 mg, enoxaparine 30 mg intravenously and prasugrel 60 mg upon FMC. The bivalirudin infusion (a bolus of 0.75 mg/kg followed by an infusion of 1.75 mg/kg/h) should be started in the catheter laboratory. After PCI, aspirin 100 mg daily should be continued indefinitely and prasugrel 10 mg daily for 12 to 15 months. A lower prasugrel dosage of 5 mg daily, or clopidogrel 75 mg daily instead of prasugrel, is recommended for patients weighing under 60 kg or aged over 75 years. Those with a previous stroke or transient ischemic attack are recommended to continue with clopidogrel 75 mg daily. When switching to clopidogrel, it is advised to load the new drug at 300–600 mg the day after prasugrel loading.

In order to assess the efficacy and safety of the new antithrombotic regimen, we examined all acute STEMI patients treated with pPCI in the Meilahti hospital between 1 January 2011 and 30 April 2012. The patients were prospectively collected into a local STEMI registry. All hospital files regarding the index STEMI hospitalisation and the following 30-day period were searched to receive detailed data on patients risk profile, treatments and clinical outcome. Mortality data was available for all patients from the National Population Register Centre. This was a registry study, which did not need an approval from the ethics committee. An informed consent was not needed; no contact to the patient was taken in this registry study and the data was anonymized and de-identified prior to analysis. The study protocol was approved by the Helsinki-Uusimaa Hospital District. The study was performed in accordance with the Declaration of Helsinki.

The inclusion criteria were acute STEMI treated with pPCI within the first 12 hours after symptom onset. The criteria for a STEMI diagnosis were acute chest pain (or equivalent) and 1) ST elevations of ≥ 2 mm (≥1.5 mm for women) in at least two of the leads V1–3, or 2) ST elevations of ≥1 mm in at least two other leads (V4–6, V8–9, V4R, I, aVL, II, III, aVF), or 3) a new left bundle branch block in ECG. Patients who received fibrinolysis were excluded from the study.

### Definitions of study endpoints

The primary endpoint was major bleeding within 30 days. Bleedings were classified and reported according to GUSTO and TIMI criteria [16, 17]. The secondary endpoints were 1) a composite of major adverse cardiovascular events (MACE) consisting of cardiovascular death, non-fatal myocardial infarction, non-fatal stroke and a new target vessel revascularisation procedure, and 2) all-cause mortality at 30 days. Myocardial infarction was defined according to current international guidelines [18]. Stroke was defined as any focal neurological deficit of ischaemic or haemorrhagic origin lasting for longer than 24 hours.

### Statistical analysis

The follow-up of the acute STEMI patients who received the guideline-defined adjuvant treatment was analysed primarily without a control group. Secondarily, a comparison with pPCI patients who had received other adjuvant treatment was performed. Continuous variables are described using medians, means and standard deviations (SD). Categorical variables are described with absolute (number) and relative (percentage) frequency distribution. Statistical analyses across the groups were done with a chi-square test for categorical variables and ANOVA for continuous variables. A Cox regression survival analysis was performed for MACE using age, sex, access site, thrombectomy, antithrombotic treatment as well as GRACE and CRUSADE scores as covariates. IBM SPSS Statistics version 19.0 was used for all analyses.

## Results

In total, 390 acute STEMI patients fulfilling the study criteria were identified within the study period. Ninety-nine patients (25 %) received the complete guideline-defined antithrombotic treatment with aspirin, a bolus of i.v. enoxaparine and a loading dose of prasugrel at presentation followed by a bivalirudin infusion at the catheter laboratory (protocol-treated group). The remaining 291 patients received some other combination of antithrombotic agents (non-protocol treated group).

The patients in the protocol-treated group were significantly younger and less likely to be on warfarin treatment at presentation than the patients in the non-protocol-treated group (Table 1). There was a trend towards a higher prevalence of diabetes and prior coronary artery bypass operations in the non-protocol-treated group. Smoking was more common in the protocol-treated group. Other cardiovascular risk factors or prior cardiovascular disease did not vary significantly across the groups (Table 1). At presentation, according to the GRACE and CRUSADE score calculations, the predicted risk of short- and long-term adverse cardiovascular events and in-hospital bleeding events was significantly higher in the non-protocol-treated group (Table 2). The proportion of patients presenting with acute heart failure was also significantly higher in the non-protocol-treated group.

**Table 1:** Baseline characteristics, cardiovascular risk factors, prior cardiovascular diseases, revascularisations and medications in the protocol and non-protocol treated groups

	Protocol	Non-protocol	*p*
	*n* = 99	*n* = 291	
Mean age, years, mean (SD)	59.5 (14)	66.1 (14)	<0.001
Age > 75 years, *n* (%)	11 (11.1)	97 (33.3)	<0.001
Male sex, *n* (%)	74 (74.7)	198 (68.0)	0.210
Diabetes, *n* (%)	13 (13.1)	64 (22.0)	0.056
Current smoker, *n* (%)	50 (50.5)	95 (32.6)	0.001
Hypertension, *n* (%)	49 (49.5)	158 (54.3)	0.408
Dyslipidaemia, *n* (%)	39 (39.4)	123 (42.3)	0.616
Renal dysfunction^a^, *n* (%)	3 (3.0)	11 (3.8)	0.729
Peripheral vascular disease, *n* (%)	4 (4.0)	14 (4.8)	0.752
Previous myocardial infarction, *n* (%)	9 (9.1)	32 (11.0)	0.593
Previous stroke, *n* (%)	4 (4.0)	21 (7.2)	0.265
Previous CABG, *n* (%)	1 (1.0)	15 (5.2)	0.073
Previous PCI, *n* (%)	10 (10.1)	39 (13.4)	0.392
Aspirin, *n* (%)	21 (21.2)	81 (27.8)	0.195
P2Y12-receptor inhibitor, *n* (%)	2 (2.0)	11 (3.8)	0.399
Warfarin, *n* (%)	2 (2.0)	37 (12.7)	0.002

*CABG* coronary artery by-pass intervention, *PCI* percutaneous coronary intervention

^a^estimated glomerular filtration rate <60 ml/min/1.73 m^2^

**Table 2:** Clinical characteristics at presentation, coronary angiography and revascularisation procedures in the protocol and non-protocol treated groups

	Protocol	Non-protocol	*p*-value
	*n* = 99	*n* = 291	
Anterior STEMI, *n* (%)	43 (43.4)	138 (47.4)	0.492
GRACE score, mean (SD)	112 (31)	124 (36)	0.002
CRUSADE score, mean (SD)	21 (13)	28(16)	<0.0001
Killip class > I, *n* (%)	8 (8.1)	62 (21.5)	0.003
Killip class IV, *n* (%)	2 (2.0)	13 (4.5)	0.270
eGFR ml/min, mean (SD)	103 (40)	90 (41)	0.009
Hemoglobin g/l, mean (SD)	135 (15)	133 (18)	0.283
Weight kg, mean (SD)	81 (16)	80 (17)	0.644
Angiography			
Radialis access, *n* (%)	33 (33.3)	77 (26.6)	0.196
3VD, *n* (%)	14 (14.1)	56 (19.2)	0.253
LMD, *n* (%)	2 (2.0)	16 (5.5)	0.154
TIMI flow grade 0–1, *n* (%)	68 (70.1)	119 (57.2)	0.031
TIMI flow grade 3, *n* (%)	15 (15.5)	58 (27.9)	0.018
Normal findings or modest coronary artery disease	0 (0)	46 (16.0)	<0.0001
PCI, *n* (%)	99 (100)	205 (70.4)	<0.0001
Use of stents (of all PCI), *n* (%)	91 (91.9)	187 (91.2)	0.838
Use of DES (of stents), *n* (%)	13 (14.3)	24 (12.8)	0.738
Thrombectomy (of all PCI), *n* (%)	53 (53.5)	68 (33.2)	0.001
TIMI flow grade 0–1 post PCI, *n* (%)	0 (0)	8 (3.8)	0.053
TIMI flow grade 3 post PCI, *n* (%)	89 (91.8)	182 (85.8)	0.143
CABG, *n* (%)	2 (2.0)	17 (5.8)	0.127

*eGFR* glomerular filtration rate estimated by Cockcroft-Gault formula, *3VD* three vessel coronary artery disease, *LMD* left main coronary artery disease, *LAD-PCI* percutaneous coronary intervention to left anterior descending artery, *DES* drug-eluting stent, *CABG* coronary artery bypass graft surgery

Sixteen percent of patients in the non-protocol-treated group had normal coronary arteries or only modest coronary artery lesions, whereas all patients in the protocol-treated group had significant coronary artery disease. Consequently, normal coronary artery flow before PCI was more frequent in the non-protocol-treated group (Table 2). However, the prevalence of left-main or tree-vessel disease did not vary significantly across the groups. A percutaneous coronary intervention was performed more often in the protocol-treated group. The use of bare metal or drug-eluting stents among the PCI-treated patients did not vary significantly across the groups. However, thrombectomy was more common in the protocol-treated group. There were no significant differences in post-PCI coronary artery flow between the groups. The median total ischaemic time (delay from symptom onset to PCI) was 300 min. and 264 min. in the protocol-treated and non-protocol-treated groups, respectively (*p* = 0.165 between the groups).

The use of glycoprotein-inhibitors was uncommon in the protocol-treated group, whereas they were used in approximately one quarter of the patients in the non-protocol-treated group (Table 3). Clopidogrel was used frequently in the non-protocol-treated group.

**Table 3:** Antithrombotic treatment in the protocol (*n* = 99) and non-protocol (*n* = 291) treated groups

	Protocol, *n* (%)	Non-protocol, *n* (%)	*P*-value
First medical contact			
Aspirin	99 (100)	257 (88.3)	<0.0001
Prasugrel loading dose	99 (100)	139 (47.8)	<0.0001
Clopidogrel loading dose	1(1.0)	116 (39.9)	<0.0001
Enoxaparin bolus i.v.	99 (100)	192 (66.0)	<0.0001
Glycoprotein IIb/IIIa inhibitor	0 (0)	15 (5.2)	0.021
Catheter laboratorio			
Bivalirudin	99 (100)	36 (12.4)	<0.0001
Glycoprotein IIb/IIIa inhibitor	6 (6.1)	79 (27.1)	<0.0001
Enoxaparin i.v.	9 (9.1)	56 (19.2)	0.019
Unfractionated heparin	0 (0)	7 (2.4)	0.119

Bleeding events were rare in the overall population, and their occurrence did not differ between the groups (Table 4). None of the patients suffered intracranial or fatal bleedings during the study period. Major adverse cardiac events and deaths were observed more frequently in the non-protocol-treated group, but the difference across the groups was not statistically significant (Table 4). In the multivariable Cox analysis for MACE, high GRACE and CRUSADE scores were the only variables associated with worse prognosis (data not shown).

**Table 4:** Major adverse cardiac events, mortality and bleeding events at 30 days in the protocol (*n* = 99) and non-protocol (*n* = 291) treated groups

	Protocol	Non-protocol	*p*-value
	*n* (%)	*n* (%)	
MACE	6 (6.1)	31 (10.7)	0.178
Cardiovascular death	4 (4.0)	26 (8.9)	0.114
Non-fatal myocardial infarction	0 (0)	1 (0.3)	0.559
Non-fatal stroke	1 (1.0)	4 (1.4)	0.781
Target vessel revascularisation	1 (1.0)	3 (1.0)	0.986
Death from any cause	5 (5.1)	28 (9.6)	0.158
Severe GUSTO bleeding	0 (0)	3 (1.0)	0.311
Mild or minor GUSTO bleeding	3 (3.0)	6 (2.1)	0.579
Major TIMI bleeding	0 (0)	1 (0.3)	0.559
Minor or minimal TIMI bleeding	2 (2.0)	4 (1.4)	0.652

At discharge, prasugrel was used in 77 and 26 % of the patients in the protocol-treated and non-protocol-treated groups, respectively, (*p* < 0.0001 between the groups) and clopidogrel in 17 and 46 % of the patients, respectively (*p* < 0.001). Statins were used in 94 and 82 % (*p* = 0.004), beta blockers in 89 and 78 % (*p* = 0.04) and angiotensin convertase inhibitors or angiotensin II receptor blockers in 81 and 65 % of the patients (*p* = 0.003), respectively.

## Discussion

### Bleeding events

In this paper, we report the results on STEMI patients treated with a novel antithrombotic regimen combining aspirin, an i.v. enoxaparine bolus and a prasugrel loading dose at presentation, followed by bivalirudin infusion at the catheterisation laboratory. To the best of our knowledge, no previous study has used the same adjuvant therapy. Our principal finding is that the administration of the new regimen is safe. None of the protocol-treated patients suffered a severe bleeding event during the 30-day follow-up. The incidence of mild to moderate bleeding events was only 3 % in that group. This outcome was favourable compared to the CRUSADE-score-based estimation of 5.5 % in-hospital incidence of bleeding events in the protocol treated group. On the other hand, the incidence of severe bleeding events was low also in the non-protocol-treated group as well, and there were no significant differences in bleeding events between the groups. Therefore, our findings support the concept that bivalirudin can be combined to low-dose low-molecular heparin without increasing bleeding complications.

The safety of bivalirudin compared to heparin plus GP IIb/IIIa-inhibitors in pPCI-patients is supported by two major trials: HORIZONS-AMI and EUROMAX [12, 13]. The incidence of net adverse and severe bleeding events was reduced with bivalirudin in both trials. In HORIZONS-AMI, bivalirudin was compared to heparin plus GP-inhibitors with almost all patients having a clopidogrel loading at the time of admission. Heparin was given in 68 % in the bivalirudin group. In the Euromax study, 51 % of the patients had a clopidogrel loading as soon as possible and the rest received either prasugrel (30 %) or ticagrelor (19 %). Heparin was used in only 2.2 % in the bivalirudin group.

### Adverse events

None of the protocol-treated patients suffered from stent thrombosis or recurrent myocardial infarction. The non-protocol group presented one stent thrombosis and one myocardial infarction within a month. The incidence of MACE and all-cause death in the protocol-treated group was 6.1 and 5.1 percent, respectively. These numbers are comparable to MACE and mortality figures recently published based on the French and Swedish STEMI registries [19, 20]. From this perspective, the new anti-thrombotic regimen also seems to be efficient.

The non-protocol group presented with a higher risk profile (older age, warfarin usage, higher GRACE and CRUSADE scores, higher Killip class at presentation, worse renal function) than those treated with the new protocol. However, the number of endpoints did not differ between the treatment groups, which is probably due to the low number of adverse events. The Cox regression analysis presented the GRACE and CRUSADE scores – which integrate patient-related information from several angles – as the only variables predicting poor prognosis.

The rate of early stent thromboses was increased with bivalirudin in both the HORIZONS-AMI and EUROMAX trials. This may be partly due to an inconsistent action of clopidogrel used widely in both trials. Importantly, in HORIZONS-AMI, those patients who had received the heparin bolus prior to randomisation had a lower risk of stent thrombosis than those without pre-randomisation heparin [21]. In EUROMAX, only 2 % of the bivalirudin treated patients were protected by heparin. However, in our study, the usage of prasugrel and enoxaparine with bivalirudin might have protected patients against stent thromboses.

The recent HEAT-PPCI study randomised STEMI patients to receive either bivalirudin or heparin infusion, with provisional GP IIb/IIIa inhibitors used in only 13-15 % of the patients in each group [14]. Only 11 % received clopidogrel, as prasugrel (27 %) and ticagrelor (62 %) were clearly more common. The rate of major adverse events during 28 day follow-up was significantly higher with bivalirudin mainly due to increased rate of stent thromboses. The bleeding rates did not differ across the groups. This might be due to low usage of GP IIb/IIIa inhibitors also in the heparin arm. Of note, the patients in the bivalirudin group were not protected with either heparin or enoxaparine.

Our hypothesis was that combining pre-hospital enoxaparine and prasugrel with bivalirudin during the pPCI procedure might balance the risk of bleeding and early stent thromboses. The observed low incidence of bleeding events and the lack of stent thromboses and recurrent myocardial infarctions in the protocol-treated patients support the hypothesis. We replaced heparin, which was widely used in the bivalirudin arm in the HORIZONS-AMI trial, by a low dose of enoxaparine at FMC without any evident rise in bleeding events.

### Adoption of the new protocol

The rate of adoption of the new local guidelines in daily practice was clearly lower than we expected. The deviations were mostly due to the omission of prasugrel and, particularly, bivalirudin. Firstly, only half of the patients in the non-protocol-treated group received prasugrel at FMC. This might reflect the fact that we started the survey period relatively soon, two months after the guideline implementation. Patients were referred to our pPCI centre from a relatively large area with different emergency medical system organisations, a setting that may have compromised the early guideline implementation process. Secondly, the utilisation of bivalirudin in the catheter laboratory was low in the non-protocol-treated group. Operators seemed to prefer to see the angiographic result before deciding whether to use bivalirudin. This policy is reflected as a higher incidence of normal angiographic findings in the non-protocol-treated groups.

### Limitations

We observed unexpectedly low bleeding rates in both treatment groups. Despite the differences in GRACE and CRUSADE scores the rate of adverse events did not differ across the groups. These findings might be related to the relatively small sample size and the non-randomised trial design, which are limitations to our study. Another limitation is the low penetration of the guidelines in daily practice, leaving open questions on the usability of the new regimen in unselected patient populations.

## Conclusions

The present study offers preliminary findings on a new antithrombotic regimen combining low-dose enoxaparine, prasugrel and bivalirudin in STEMI patients. The data are promising and suggest that the regimen is both safe and efficient, but the results need to be confirmed in randomised studies.

## Abbreviations

ACS, acute coronary syndrome; CABG, coronary artery by-pass graft surgery; FMC, first medical contact; Gp-inhibitor, glycoprotein receptor inhibitor; MACE, major adverse cardiovascular event; PCI, percutaneous coronary intervention; STEMI, ST-segment elevation myocardial infarction
